# Exploring the impact of China-Europe Railway Express on the urban-rural income gap

**DOI:** 10.1016/j.heliyon.2023.e17571

**Published:** 2023-06-28

**Authors:** Lei Zhang, Jianghong Wan, Amar Razzaq, Qing Zhang, Ling Zhou, Sahar Erfanian

**Affiliations:** a College of Economics and Management, Huazhong Agricultural University, No. 1 Shizishan Street, Hongshan District, Wuhan, 430070, China; b College of Humanities and Social Sciences, Huazhong Agricultural University, No. 1 Shizishan Street, Hongshan District, Wuhan, 430070, China; c Business School, Huanggang Normal University, No. 146 Xinggang 2nd Road, Huanggang, 438000, China; d Business School, Hunan First Normal University, Changsha, 410205, China; e School of Foreign Studies, Hunan University of Technology and Business, No.569 Yuelu Avenue, Yuelu District, Changsha, 410205, China

**Keywords:** CRexpress, Urban-rural income gap, International trade, Industrial development, Labor mobility

## Abstract

The China-Europe Railway Express (CRexpress) has established a new land transportation route between Asia and Europe as part of China’s westward expansion. The resulting trade promotion effect has the potential to improve industrial development and factor flows, ultimately leading to a reduction in the income disparity between urban and rural areas in cities that use the CRexpress. The impact of the CRexpress on income disparities between urban and rural areas in cities that use the service is of particular interest, as the empirical evidence on the relationship between international trade and these disparities is inconsistent. Using a difference-in-differences model and macro panel data, this study found that the CRexpress significantly narrowed the urban-rural income gap in cities where it was operational, and that this effect had a spillover effect on nearby cities. However, the magnitude of this effect decreased with distance. The mechanism analysis indicated that the CRexpress narrowed the income gap by promoting secondary industry development, but this effect varied significantly by region, with pronounced effects in eastern coastal cities and less pronounced effects in inland cities in the central and western region. The study suggests that local governments in these regions should focus on improving the institutional environment and providing industrial support to promote industrial transfer in order to narrow the urban-rural income gap and promote overall economic development.

## 1 Introduction

With China’s “growth miracle”, the issue of income polarization and regional imbalance has become more prominent. One particularly pressing concern is the significant gap between urban and rural incomes, which has consistently dominated the overall income gap in China [[1], [2], [3], [4], [5]]. The income ratio between urban and rural residents in China increased from 2.57 in 1978 to a high of 3.33 in 2007, and while it has moderated since then, it remained high at 2.56 in 2020. This large urban-rural income disparity is unsustainable for macroeconomic growth and poses a threat to social stability. The role of international trade in industrial development and wealth distribution is critical among the various factors that affect the urban-rural income gap. Scholars have conducted extensive research in this area. Following China’s entry into the WTO in 2001, the most significant change in the realm of international trade has been the establishment of the “Belt and Road Initiative”. As the flagship initiative of this project, CRexpress provides a safe and efficient land transportation channel for China-EU trade, which has resulted in a substantial boost in China-EU trade growth. Furthermore, it has played a crucial role in driving the development of industries along the CRexpress route [6]. Therefore, a question worth considering is whether the opening of the CRexpress has an impact on the urban-rural income gap in China. If this impact exists, what are the corresponding mechanisms and heterogeneity characteristics? Clarifying the above problems has important theoretical and practical significance for accurately assessing whether the CRexpress can become a new force to improve the urban-rural income distribution pattern.

There is a certain theoretical basis and practical experience for the impact of international trade on the urban-rural income gap. In theory, international trade will increase the prices of a country’s richer factors and decrease the prices of scarcer factors, while China has abundant low-skilled labor (rural surplus labor), but high-skilled labor is relatively scarce (urban labor), so international trade is conducive to narrowing the urban-rural income gap. In terms of China’s practice, since the reform and opening up, international trade has played a key role in promoting China’s economic growth, industrial development and income level. However, China’s gradual opening-up policy has led to different degrees of opening up between urban and rural areas and between regions. The income distribution effects caused by trade expansion are also significantly different. Due to the convenience of being close to ports, the eastern coastal areas have become the main areas where China’s export-oriented industries are concentrated, thus creating a large number of jobs and absorbing a large number of rural surplus labor. Therefore, the eastern coastal areas are the areas with the highest degree of industrialization in China and the smallest urban-rural income gap [7]. Based on the above reasoning, this paper posits that the CRexpress’s trade promotion effect has an impact on the urban-rural income gap in China. Firstly, the CRexpress has rapidly developed since its inception, reshaping China’s economic geography. China and the European Union’s highly complementary industries have led to a trade volume of over US $828.11 billion in 2021, with the CRexpress transporting goods worth over US $200 billion. The CRexpress has bridged the gap in logistics endowment and effectively facilitated the “portalization” of inland cities. Secondly, the CRexpress has stimulated the development of export-oriented industries in inland areas and generated employment opportunities, particularly for the secondary industry, which may serve as a potential channel for the CRexpress’s impact on the urban-rural income gap. Additionally, China’s vast territory and regional disparities in economic development, institutional environment, and resource endowments will influence the CRexpress’s utilization in different regions, making it possible to discuss regional heterogeneity. Finally, the CRexpress’s rapid development provides a quasi-natural experiment for studying the impact of international trade on the urban-rural income gap in China. The CRexpress involves the interests of many countries in Eurasia, and requires multiple rounds of consultations between countries on problems such as export customs transit supervision and customs clearance facilitation before opening. There is great uncertainty about the opening time and the opening city, which meets the requirements of the quasi-natural experiment to build treatment and control groups.

The objective of this paper is to explore the impact of CRexpress on China’s urban-rural income gap, as well as the mechanism and heterogeneity behind it, by constructing a more general multi-period DID model and using regional-level panel data. The study found that: First, on average, compared with the cities that did not open the CRexpress, the urban-rural income gap in the cities that opened the CRexpress narrowed by 3.86%. This effect has a radiation effect on the cities around the cities that opened the CRexpress, and decreases with the increase of distance; Second, the narrowing of the urban-rural income gap by the CRexpress is mainly achieved by promoting the development of the secondary industry. The opening of the CRexpress has promoted the development of the secondary industry and provided more non-agricultural jobs for migrant workers, which is conducive to narrowing the urban-rural income gap; Third, the effect of CRexpress on narrowing the urban-rural income gap has significant regional heterogeneity, and the effect on the eastern coastal areas is greater than that on the central and western inland areas, and the institutional environment has played a positive moderating role in this process; Fourth, in terms of the income structure of urban and rural residents, the narrowing of the urban-rural income gap by CRexpress is mainly achieved by affecting the transfer of net income.

This paper makes several contributions to the existing literature. Firstly, it studies the urban-rural income gap in China from the perspective of CRexpress based on the “Belt and Road Initiative” strategy, which is a novel approach. Secondly, this paper takes into account the unique features of the CRexpress such as “divided cities, divided time periods, and different frequencies”, and employs a multi-period DID model to effectively address endogeneity issues. Additionally, it utilizes data after 2003 to remove the confounding factor of China’s entry into the WTO, thus providing a more accurate assessment of the policy impact of CRexpress. Thirdly, this paper conducts a comprehensive analysis of the channels and mechanisms through which CRexpress affects the urban-rural income gap, taking into account regional heterogeneity, and presents extensive policy recommendations.

## 2 Literature review

To effectively narrow the urban-rural income gap, we must first understand the main reasons for the widening of the urban-rural income gap. From the existing literature, scholars' research on the factors affecting the urban-rural income gap can be roughly divided into two categories: endogenous factors and exogenous factors. Among them, endogenous factors mainly refer to factors related to economic growth, such as the degree of urbanization [8], the level of financial development [9], industrial structure, human capital [10], Foreign direct investment [11], etc. Exogenous factors mainly refer to some policy factors, such as the government’s biased policies [12], fiscal expenditure system [13], institutional environment [14,15], household registration system [16], etc. These studies provide important inspiration for this paper. With the continuous and in-depth advancement of globalization, international trade is often used by scholars to explain the urban-rural income gap, which is exactly what this article is about.

The literature closely related to this article can be roughly divided into three categories: First, from the perspective of globalization, some scholars believed that globalization would narrow a country’s income gap, which was supported by experience in countries where the income gap had been narrowed after opening to the outside world [[17], [18], [19], [20]], but these studies are at the national level and cannot observe differences in different regions within a country. Further, Wei S J and Wu Y [21] applied the geography-based instrumental variable method to overcome the possible endogenous problem of trade opening, and examined the urban-rural income ratio of 100 cities in China from 1988 to 1993, and found that there was a negative relationship between trade opening and the urban-rural income gap. In terms of influence mechanism, Yuan Dongmei [7] found that the expansion of foreign trade would lead to changes in commodity structure and promote the development of China’s labor-intensive industries, thereby narrowing the urban-rural income gap in China, but it couldn’t solve the endogenous problem well. Second, some studies have suggested that international trade increases disparities in returns to skills and education, thereby widening income disparities [22], and there is empirical support in some transition economies [23]. Helpman [24] used within-country micro data to demonstrate that international trade increased wage inequality. But these studies lack attention to the urban-rural income gap. Third, some literatures have believed that the relationship between foreign trade and the urban-rural income gap is uncertain. Foreign trade has employment expansion effects and employment quality bias effects, and the impact on the urban-rural gap depends on the combination of these two opposite effects. The effects of the two are different in different periods, so the total effect is uncertain [[25], [26], [27]]. Overall, domestic and foreign studies on the impact of foreign trade on the urban-rural income gap have not reached a consistent conclusion, mainly due to differences in sample coverage and time and space, hence a detailed discussion of heterogeneity is particularly important.

Existing research is of great significance for understanding the link between international trade and urban-rural income gap, but there are the following shortcomings: First, the “Belt and Road Initiative” strategy represented by the CRexpress has reshaped China’s economic geography to a certain extent and is an important milestone in China’s opening to the west. Unfortunately, no scholars have paid attention to the impact of the CRexpress on the urban-rural income gap; Second, some studies have not solved the endogenous problem well; Third, previous studies have well demonstrated the impact and mechanism of foreign trade on the urban-rural income gap, but the heterogeneity analysis is insufficient. This paper is committed to making improvements in these aspects.

## 3 Concept definition and theoretical analysis

### 3.1 Introduction to CRexpress (China-Europe Railway Express)

**Fig. 1:**
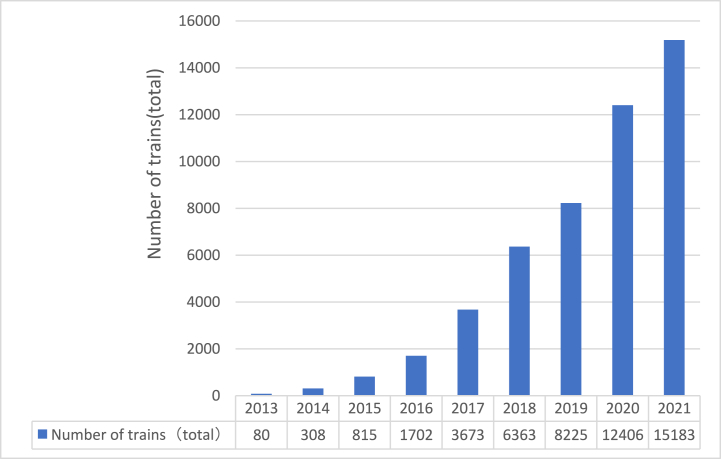
The development of CRexpress (The data comes from the “China-Europe Railway Express Development Report (2021)”).

The China-Europe Railway Express (CRexpress) is a railway container freight train operated by the China Railway Corporation. It runs between China and Europe and the countries along the 'Belt and Road Initiative,’ following fixed lines, trains, schedules, and full timetables. The CRexpress connects the Silk Road Economic Belt in the north with the 21st Century Maritime Silk Road in the south, and serves as a key component of China’s strategy for further opening up to the west. It has seen significant growth since its inception in 2011 (see Fig. 1).

According to the 'China-Europe Railway Express Development Report (2021)', trade between China and the European Union reached $828.11 billion in 2021, with goods transported by the CRexpress accounting for more than $200 billion of this total. The CRexpress has shortened the 'space-time distance’ between China and Europe and reduced the cost of international trade, providing a window for China’s westward opening and helping to compensate for the inland areas' historical disadvantage in a maritime-led opening to the outside world.

**Fig. 2:**
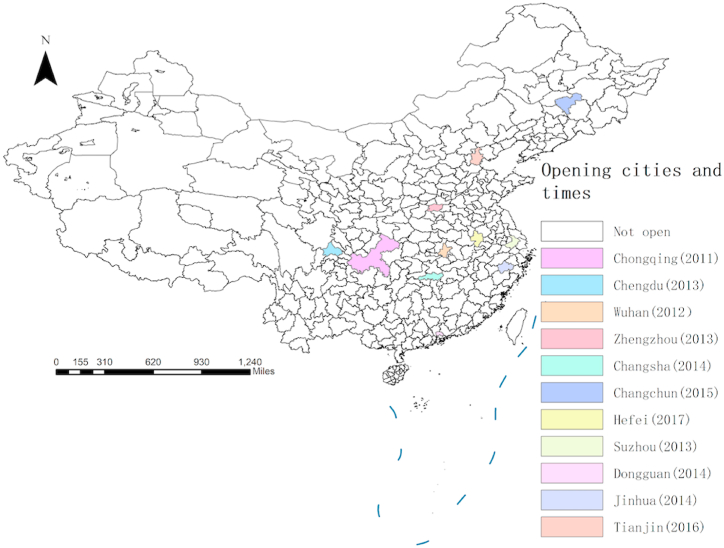
Cities that have normalized the opening of CRexpress.

Fig. 2 shows the cities have normalized the opening of CRexpress. The opening of the CRexpress provides an ideal quasi-natural experiment for studying the impact of international trade on the urban-rural income gap. Firstly, the opening of the CRexpress is determined by government decision-making and involves the interests of many countries along its route. Before opening, multiple rounds of consultation are required on issues such as railway freight transfer, customs clearance facilitation, and export supervision. The city, time, and frequency of the opening of the CRexpress are unpredictable, meeting the exogenous assumption of policy impact. Secondly, the opening of the CRexpress has the characteristics of a city division and gradual opening. It uses the existing railway system rather than redesigning and constructing it, meeting the randomness conditions required for the construction of control groups and treatment groups in quasi-natural experiments.

Table 1 presents the basic information for some cities that have normalized the operation of CRexpress. As compared to the eastern regions, the central and western regions have clear advantages in terms of transportation distance and one-way time in terms of cost and efficiency. The goods transported by CRexpress are primarily high-value-added products such as electronics, mechanical equipment, auto parts, and high-end clothing. The primary goods transported by CRexpress suggest that industries having benefited more from the CRexpress opening are concentrated in the secondary sector, which aligns with China’s current endowment advantage in the global industrial chain, particularly in labor-intensive, high-tech products. This information suggests that the impact of CRexpress on the urban-rural income gap may be closely related to the development of the secondary sector. In the following sections, this paper will further discuss its theoretical mechanism.

**Table 1:** Part of cities that have normalized the operation of CRexpress and main commodities.

Region	City (year)	Main commodities (China →Europe)	Distance (Km)	Time (day)
Western	Chongqing (2011)	Laptops, LCD panels, mechanical equipment, auto parts, biomedicine	11179	16
	Chengdu (2013)	Electronics, auto parts, mechanical equipment	9826	11
Central	Wuhan (2014)	Electronic products, optical cables	10863	18
	Zhengzhou (2013)	Electronic products, mechanical equipment, high-end clothing	10245	15
	Changsha (2015)	Electronic products, mechanical equipment, auto parts	11800	17
	Hefei (2017)	Solar PV, Sensors, Robotics	10873	16
Eastern	Suzhou (2013)	Electronic products, mechanical equipment, clothing	11200	18
	Dongguan (2014)	Electronic products, light industrial products	13650	20
	Jinghua (2014)	Electronic products, daily chemical products, hardware tools, motorcycles	11000	22

### 3.2 Theoretical analysis on CRexpress and urban-rural income gap

The theory of international trade and residents' income can be traced back to the neoclassical H–O model, which posits that capital and investment tend to flow to areas with low factor prices and labor will flow to areas with higher wages, leading to a narrowing of the income gap. This model, as well as the FPE theorem and S–S theorem developed on its basis, typically examine the relationship among trade, factor prices, and factor rewards. According to this view, a country exports factor-intensive products with a comparative advantage and imports scarce production factor-intensive products. As a result, foreign trade increases the prices of a country’s more abundant factors and decreases the prices of less scarce factors.

The CRexpress has a trade promotion effect. The 'trade growth effect’ of the new economic geography provides theoretical support for the CRexpress to promote the opening of regional trade growth. Improving transportation infrastructure can effectively reduce transportation costs, indirectly improving enterprise production efficiency [28] and increasing trade volume, as confirmed by empirical studies on the CRexpress [29,30].

To simplify the theoretical analysis, Chinese scholars often divide the labor force into low-skilled and high-skilled labor, with the former referring to rural transfer labor with lower human capital and the latter referring to urban residents with higher human capital [31]. In China, high-skilled labor is relatively scarce and low-skilled labor is relatively abundant [25]. Therefore, the trade promotion effect brought about by the CRexpress will contribute to raising the wages of low-skilled labor, primarily rural transfer labor, and inhibiting the wages of high-skilled labor, primarily urban residents, resulting in a narrowing of the urban-rural income gap. Based on this analysis, this paper proposes:

Hypothesis 1 The CRexpress is conducive to narrowing the urban-rural income gap in cities with CRexpress.

As previously mentioned, the CRexpress primarily transports goods from the manufacturing industry, which suggests that the CRexpress is unlikely to have a significant impact on the urban-rural income gap through the primary industry. However, the expansion of Sino-European trade caused by the CRexpress promotes the growth of labor-intensive industries in China, particularly the secondary industry of manufacturing. These industries often employ rural labor, making them the primary beneficiaries of the employment expansion caused by the opening of CRexpress. This can help narrow the income gap between urban and rural residents. The expansion of the secondary industry may also lead to growth in the related tertiary industry, such as transportation, warehousing, finance, and business services, but it is unclear if this has an indirect effect on the urban-rural income gap. Based on this analysis, we propose the following:

Hypothesis 2 The effect of CRexpress on narrowing the urban-rural income gap is achieved through the expansion of the secondary industry.

The Westward opening up of China represented by the CRexpress has facilitated the transfer of industries and factors to the west, highlighting the importance of considering the flow of factors and the related institutional environment. China’s economy is characterized by an urban-rural dual structure, with significant differences in economic development and institutional environments across regions. According to Lewis’s dual economic theory, the root cause of the urban-rural income gap in developing countries is the much higher labor productivity of the modern urban sector compared to the traditional rural sector. As rural surplus labor moves to cities to work in non-agricultural industries, the surplus labor force will eventually be absorbed, leading to convergence in labor productivity between the industrial and agricultural sectors and narrowing of the urban-rural income gap. Ranis-Fei model further divided the flow of labor to the industrial sector into three stages: the stage of unlimited supply of labor, the stage of limited supply of labor, and the stage of dual economic integration [32].

Since the 1990s, a large number of surplus rural laborers from the central and western region have poured into eastern cities for employment, which has increased the household income of rural residents and eased the pressure of rising urban wages in the eastern regions. This cross-regional flow of labor has greatly extended the time window for the eastern regions' demographic dividend, enabling the eastern regions' export-oriented economy to develop rapidly for a long time. It can be said that the first stage of dual economic development (the stage of unlimited supply of labor) has been well interpreted in China.

China’s accession to the WTO in 2001 further accelerated the transfer of surplus rural labor, and many studies have shown that the country reached the first Lewis inflection point around 2004, marking the transition to the second stage of the dual economy, the “limited labor supply stage” [33]. In this stage, the proportion of the industrial sector increased, the agricultural sector shrank relatively, the growth rate of rural transfer labor slowed down, and the wages of the urban industrial sector rose, in line with theoretical expectations. However, contrary to theoretical expectations, China’s industrial and agricultural labor productivity has not shown a convergence trend. The country entered the late stage of industrialization from 2010 to 2014 and was expected to achieve industrialization by 2020, but a significant number of rural laborers remained “stranded” in the countryside [34,35]. This suggests that China has entered the Lewis turning point “prematurely” before the rural labor force has been fully transferred.

According to Lewis’s dual economic theory, the ideal condition for the flow of factors is a “free flow” in which there are no barriers to the movement of labor and other resources between urban and rural areas and between regions. However, in reality, there are varying degrees of market segmentation between regions in China (Local protectionism), and a unified national labor market has not yet been established. There are still many tangible and intangible institutional obstacles between urban and rural areas and between regions that hinder the free flow of labor between urban and rural areas [36]. The quality of the institutional environment plays a role in determining the extent to which the market mechanism can function effectively, meaning that the flow of regional factors is more sufficient in regions with a better institutional environment. In addition, the institutional environment also affects the transaction costs of enterprises to a certain extent. Regions with a better institutional environment have lower transaction costs for enterprises [37]. Therefore, this paper proposes:

Hypothesis 3 The institutional environment has a positive moderating effect on the effect of CRexpress on narrowing the urban-rural income gap.

**Fig. 3:**
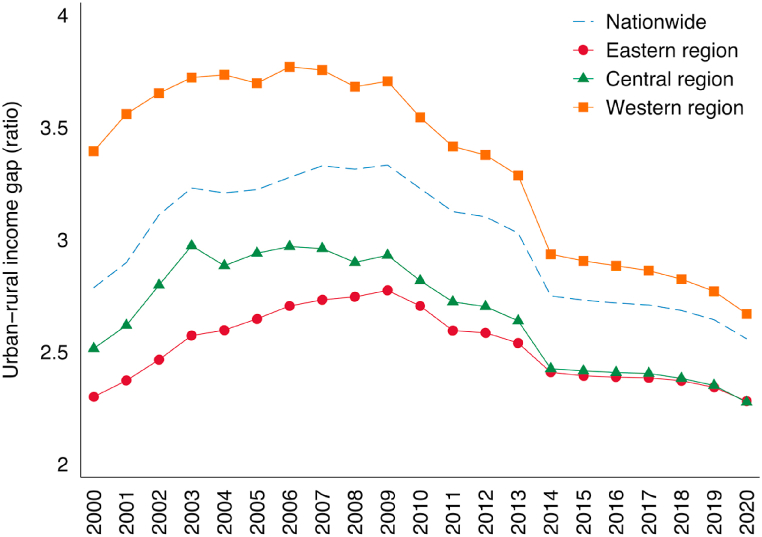
Urban-rural income gap by region.

The transportation cost effect of CRexpress on the urban-rural income gap varies by region. Trade costs, including transportation costs, are known to impact bilateral trade volume. According to the classic trade attractive force model, trade volume between two countries is proportional to their economic scale and inversely proportional to the transportation distance between them [38,39]. Within a country, domestic transportation costs can vary and impact the ability of different regions to participate in international trade. In China, the eastern region has traditionally had a transportation cost advantage due to its proximity to ports, leading to higher levels of economic development and a narrower urban-rural income gap compared to the central and western regions (as shown in Fig. 3). The transportation distance and one-way time of cities with CRexpress service, listed in Table 1, show that the central and western regions have a transportation cost and efficiency advantage in that they are closer to European market.

The opening of CRexpress helps to mitigate the disadvantage of international logistics in inland areas, allowing these regions to participate more fully in the international division of labor and improving the response speed of inland enterprises in global value chains, thereby reducing export costs for these enterprises [40]. On one hand, transportation infrastructure optimally allocates factors by reducing transportation costs [6], attracting production factors and industries to move westward, as well as leveraging the endowment advantages of labor and land in inland areas. On the other hand, improved transportation infrastructure can also promote market responsiveness and correlation [41], attracting more manufacturers and foreign capital, creating scale and agglomeration effects, reducing market segmentation, and further reducing transportation costs.

It is worth noting that, the first-mover advantage of the eastern coastal areas cannot be ignored. As is well-known, China’s reform and opening up is gradual. As the frontier of opening up, the eastern regions have accumulated great advantages. Not only is the level of economic development much higher than that of the inland regions, but also the institutional environment is better. During the sample period, the mean values of the institutional environment in the eastern, central and western regions were 8.02, 6.17 and 5.51, respectively, showing an obvious trend of high in the east and low in the west. The disadvantage of the institutional environment in inland areas will undoubtedly increase transaction costs and hinder the flow of factors, which is not conducive to the industrial development of inland areas. Namely, inland areas have both the geographical advantage of being closer to the European market and the disadvantage of having a poorer institutional environment than coastal areas, and vice versa. Therefore, the regional heterogeneity of CRexpress narrowing the urban-rural income gap depends on the combination of transportation costs (geographic distance) and transaction costs (institutional environment). Based on this analysis, the following hypothesis is proposed:

Hypothesis 4 The effect of CRexpress narrowing the urban-rural income gap is regionally heterogeneous and depends on the combination of transportation costs and transaction costs, which needs to be empirically tested.

In addition, CRexpress strengthens the function of gathering resource elements in inland areas, amplifying the “siphon effect” of the source node and the “radiation effect” of the transportation channel [42]. China’s domestic logistics and transportation network has long been complete, there may be spillover effects between cities; namely, cities that have not opened CRexpress may also use CRexpress to carry out foreign trade, which is conducive to narrowing the urban-rural income gap. Therefore, this paper proposes:

Hypothesis 5 The effect of CRexpress on narrowing the urban-rural income gap may have a radiation effect on cities surrounding those having opened CRexpress.

## 4 Materials and methods

### 4.1 Data source

This study used panel data from 250 cities and 31 provincial panels in mainland China from 2003 to 2020, excluding samples with serious data loss or outliers during the sample period. Our identification strategy (DID) relied on the absence of other policy shocks related to the opening of China-Europe trains to the west during the sample period. However, China joined the WTO in 2001. Before and after that, the domestic and foreign economic environment has undergone profound changes. In order to eliminate this confounding factor, this paper used data after 2003 to make policy identification cleaner. The prefecture-level macro data is from the “China Urban Statistical Yearbook” published during the study period, and the provincial macro data is from the CSMAR database, as well as the “China Statistical Yearbook” and “China Marketization Index Report” published during the same time frame. Data on cities that normally operate CRexpress is obtained from the official websites of the Belt and Road Initiative, the Ministry of Railways, and the China-Europe Railway. The cities included in the analysis are those that have normalized the operation of CRexpress. In addition, the National Bureau of Statistics of China implemented an urban-rural integration survey in 2013 and adjusted the definition of migrant workers' attribution and transfer net income. Therefore, this study includes panel data after 2013 as a sub-sample regression to increase the robustness of the conclusions.

### 4.2 Identification strategy and model settings

Similar to many policies in China, the opening of the CRexpress is gradual. Initially, Chongqing was used as a pilot city. The first CRexpress was opened in 2011, and then it was successively extended to other cities that had the conditions to open the CRexpress. Theoretically speaking, there are “policy effects” and time effects on the impact of the CRexpress on the urban-rural income gap. Among them, the policy effect is the impact caused by the impact of the opening of the CRexpress. However, the opening of the CRexpress has the characteristics of “divided cities, divided time periods and different frequencies”. In order to better identify the relationship between this spatiotemporal staggered event and the urban-rural income gap, this paper adopts the multi-period DID model for analysis. Specifically, considering that the “axis-width” organization and management model of the CRexpress will also have a radiating effect on the surrounding cities, and the provincial administrative departments enjoy the local administrative and legislative power in accordance with the law and are responsible for the overall management of the economic development within their jurisdiction, this article refers to Li Jia [43], all prefecture-level cities in the province where the city that has opened CRexpress is located are placed in the treatment group, and other prefecture-level cities are placed in the control group. The benchmark model in this paper is set as follows:

(1) $${gap}_{it}=\beta {CRexpress}_{it}+\delta Z_{it}+\gamma_{t}+\mu_{i}+\varepsilon_{it}$$

Among them, i represents the cities in the country, and t represents the year. The explanatory variable gap_it_ represents the ratio of urban-rural income of city i in year t (take the logarithm). The core explanatory variable CRexpress_it_ indicates whether city i will open CRexpress in year t. If it is not opened, the value will be 0, and the value will be 1 in the year of opening and subsequent years.

The coefficient β is the income effect of the opening of CRexpress on urban and rural residents that this paper is concerned about, and refers to the change of the urban-rural income gap after the opening of CRexpress compared with the cities having not opened CRexpress. If β is significantly less than 0, which means that the opening of CRexpress will help the city narrow the urban-rural income gap. Z_it_ is a series of control variables. γ_t_ is a year-fixed effect used to control for time effects that do not vary with cities. μ_i_ is an urban fixed effect used to control urban characteristics that do not change with year. ε_it_ is a random perturbation term. As a transportation infrastructure, CRexpress not only has an impact on cities with CRexpress, but also has a radiation effect on the surrounding cities, and may affect each other between neighboring cities (such as trade, investment, environment, industrial agglomeration). Therefore, considering the potential sequence correlation and heteroskedasticity, this paper adopts a robust standard error for clustering to the city level.

### 4.3 Variable setting and descriptive statistics

Dependent variable. The dependent variable for this study is the urban-rural income gap, which is calculated as the ratio of urban residents' disposable income to rural residents' disposable income. The variable is entered into the regression model in the form of a logarithm.

Core explanatory variable. The core explanatory variable in this paper is the opening of CRexpress, with a value of 1 for the year of opening and subsequent years, and 0 otherwise.

Control variables. In addition to the opening of CRexpress, other factors may also affect the urban-rural income gap. This study controls for the following variables: (1) regional economic development level (per_GDP), measured as real per capita GDP (logarithm); (2) regional industrial structure (structure_2), measured as the proportion of the secondary industry’s added value to GDP; (3) regional education level (education), measured as the proportion of the total population with ordinary college education; (4) fiscal expenditure (finance), measured as the proportion of regional fiscal expenditure in GDP; (5) foreign direct investment (FDI), measured as the amount of foreign capital actually used in the region (logarithm); (6) urbanization level (urbanization), measured as the proportion of urban permanent residents to the total population at the end of the year; (7) level of scientific and technological innovation (technology), measured as the number of regional patents granted (logarithm); (8) institutional environment (institution), measured using mainstream methods in academic circles [44] as the regional market index (market) and market segmentation index (segmentation). The calculation method is: institution = market (1-segmentation), where the market index is taken from the “China Marketization Index Report” compiled by Fan Gang and others, and the segmentation index is calculated by using the price index method [45]. Table 2 presents the descriptive statistical results of the main variables in this paper.

**Table 2:** Descriptive statistics of variables (2003–2020).

Variable	N	Mean	SD	Min	Max
gap	3265	0.920	0.201	0.259	1.717
CRexpress	3265	0.105	0.306	0	1
structure_2	3265	0.494	0.0990	0.149	0.859
urbanization	3265	0.726	0.339	0.0990	1
finance	3265	0.144	0.0800	0.0150	1.936
FDI	3265	9.713	1.768	2.485	14.15
education	3265	13.67	1.116	8.978	16.39
per_GDP	3265	3.298	2.629	0.256	29.05
technology	3265	6.293	1.656	1.792	11.42
institution	3265	6.734	1.566	2.329	11.39

## 5 Results

### 5.1 Impact of CRexpress on urban-rural income gap: benchmark regression

The results of the benchmark regression are presented in Table 3. In column (1), only the time dummy variable and the core explanatory variable of CRexpress opening are included, and the regression coefficient is negative and significant at the 1% level. Columns (2) to (7) present the results of the regressions with the stepwise addition of control variables, and all regressions have included two-way fixed effects. Despite the decrease in the regression coefficient, it remains significant at the 1% level. Using the results in column (7) as a reference, on average, the urban-rural income gap in cities that have opened CRexpress has narrowed by 3.86% compared to cities that have not. This supports Hypothesis 1. In addition, the 10.13039/100009376National Bureau of Statistics of China adjusted the statistical caliber of urban and rural residents' income in 2013. In column (8), this study includes a sub-sample of data after 2013 in the regression to improve the robustness of the conclusion. The results show that under the new statistical caliber, CRexpress is still effective in narrowing the urban-rural income gap in cities with CRexpress, and the absolute value and T value of the regression coefficient have increased.

**Table 3:** Benchmark regression results.

VARIABLES	(1)	(2)	(3)	(4)	(5)	(6)	(7)	(8)
	gap	gap	gap	gap	gap	gap	gap	gap
CRexpress	−0.0424***	−0.0390***	−0.0391***	−0.0394***	−0.0399***	−0.0398***	−0.0386***	−0.0511***
	(−3.69)	(−3.48)	(−3.51)	(−3.55)	(−3.63)	(−3.63)	(−3.58)	(−4.68)
structure_2		−0.2400***	−0.2454***	−0.2469***	−0.2143***	−0.2178***	−0.2007***	−0.5345***
		(−3.22)	(−3.50)	(−3.51)	(−2.99)	(−3.02)	(−2.90)	(−5.79)
urbanization			0.0088	0.0119	0.0120	0.0106	0.0285	−0.0537
			(0.20)	(0.27)	(0.28)	(0.24)	(0.61)	(−0.92)
finance				−0.0478	−0.0309	−0.0295	−0.0251	0.0017
				(−1.15)	(−0.81)	(−0.77)	(−0.75)	(0.07)
FDI					−0.0135***	−0.0136***	−0.0133***	−0.0003
					(−3.19)	(−3.20)	(−3.15)	(−0.11)
education						0.0053	0.0044	−0.0193**
						(0.59)	(0.50)	(−2.15)
per_GDP							0.0009	−0.0060**
							(0.23)	(−2.23)
technology							−0.0200***	−0.0204**
							(−2.92)	(−2.13)
Observations	3265	3265	3265	3265	3265	3265	3265	960
Adjusted R-squared	0.854	0.857	0.857	0.857	0.859	0.859	0.861	0.913
City FE	YES	YES	YES	YES	YES	YES	YES	YES
Year FE	YES	YES	YES	YES	YES	YES	YES	YES

Robust t-statistics in parentheses ***p < 0.01, **p < 0.05, *p < 0.1.

### 5.2 Evaluating the robustness of the effects of CRexpress on narrowing the urban-rural income gap

To ensure the validity of the difference-in-differences (DID) approach, it is necessary to establish that the urban-rural income gap in the treatment and control groups had a similar trend prior to the policy shock. This paper uses a method in which each year in the sample period is set as a dummy variable, which is multiplied by the dummy variable of the policy impact and subjected to a regression test. The previous period before the policy impact is taken as the benchmark. If the regression coefficients prior to the opening of CRexpress are not statistically significant, it means that the model satisfies the parallel trend assumption. As shown in Fig. 3, “pre2”to “pre7” refer to the regression coefficients from two to seven years before the opening of CRexpress, which are not statistically significant, indicating that there is no significant difference between the treatment and control groups. The regression coefficients for the year following the opening of CRexpress, namely “current,” “post1”, and “post2”, show a significant negative trend. This suggests that CRexpress has a positive impact on narrowing the urban-rural income gap in the cities where it has been introduced.

### 5.3 Mechanism analysis: the mediating effect of the secondary industry and the moderating effect of the institutional environment

Table 4 presents the results of the mechanism test examining the impact of CRexpress on the urban-rural income gap. Two-way fixed effects and control variables are included in columns (1) to (4). As previously mentioned, the opening of CRexpress may promote the growth of a tradable secondary industry and generate more non-agricultural jobs, which can increase the non-agricultural income of the rural surplus labor force and subsequently narrow the urban-rural income gap. This study tests Hypothesis 2 using a stepwise regression approach. In column (1), the regression of the opening of CRexpress on the proportion of the secondary industry shows a significantly positive coefficient at the 10% level. The results indicate that, compared to cities that have not opened CRexpress, the proportion of the secondary industry in the cities that have opened CRexpress has increased by 1.17%. In column (2), the regression of the opening of CRexpress on the urban-rural income gap is significantly negative, indicating that the opening of CRexpress is effective in narrowing the urban-rural income gap in the cities that have opened CRexpress. Column (3) includes the regression of the two explanatory variables of the opening of CRexpress and the proportion of the secondary industry on the urban-rural income gap. The coefficient for the opening of CRexpress is negative, and the absolute value and T value have decreased, which is consistent with expectations. This supports the conclusion that Hypothesis 2 is valid.

**Table 4:** Mediating and moderating effects.

VARIABLES	(1)	(2)	(3)	(4)
	industry-2	gap	gap	gap
CRexpress	0.0117*	−0.0418***	−0.0395***	0.1187***
	(1.82)	(−3.83)	(−3.63)	(2.71)
CRexpress × institution				−0.0189***
				(−3.28)
institution				−0.0272***
				(−3.77)
industry-2			−0.1956***	−0.1849***
			(−2.80)	(−2.66)
Observations	3218	3218	3218	3218
Adjusted R-squared	0.843	0.858	0.860	0.863
Control	YES	YES	YES	YES
City FE	YES	YES	YES	YES
Year FE	YES	YES	YES	YES

Robust t-statistics in parentheses ***p < 0.01, **p < 0.05, *p < 0.1.

Although the transportation goods of the CRexpress are mainly concentrated in industrial products, there are still many small and medium-sized cities that may rely on both agriculture and industry for their development, and industrial development may also drive the growth of related service industries. Namely, the primary industry and the tertiary industry cannot be excluded. This potential channel of influence. Therefore, this paper also examines two potential influence channels, the primary industry and the tertiary industry, and rules out this possibility. Interested readers can ask the author for detailed results.

In addition, as an international freight infrastructure, the CRexpress has typical public goods characteristics, but there are differences in its utilization in different regions. On one hand, it stems from endogenous factors such as the level of regional economic development, and on the other hand, it is also subject to the institutional environment of the region, such as the degree of marketization, business environment, legal environment, etc. Regions with a better institutional environment also have lower transaction costs, which is conducive to the development of local enterprises and attracts external capital. Therefore, regions with a better institutional environment may make full use of the CRexpress to further open up the international market and develop local industries, thereby benefiting migrant workers in the region. In order to verify the above conjectures, in column (4) of Table 4, this paper adds the interaction term between the dummy variable of the opening of the CRexpress and the institutional environment, and the regression coefficient is significantly negative at the level of 1%. This shows that the improvement of the institutional environment has strengthened the effect of narrowing the urban-rural income gap, so that Hypothesis 3 has been verified.

### 5.4 Regional heterogeneity analysis

In general, areas that are geographically further away from cities where CRexpress is available may face higher transportation costs and fewer benefits from economic agglomeration, resulting in comparatively less advantage for peripheral regions.

The results of the analysis of the impact of CRexpress on the urban-rural income gap in surrounding cities with a radius of 100–300 km are presented in Table 5. Column (1) shows that, on average, the opening of CRexpress in a city significantly narrows the urban-rural income gap in surrounding cities with a radius of 100 km by 4.55%. As the radius increases, the effect of CRexpress on narrowing the urban-rural income gap decreases, as shown in columns (2) and (3). These results support the Hypothesis 5 that the opening of CRexpress has a radiation effect on surrounding cities, with the effect decreasing with an increase in the radiation radius.

**Table 5:** Radiation effect.

VARIABLES	(1)	(2)	(3)
	radius 100 km	radius 200 km	radius 300 km
	gap	gap	gap
CRexpress	−0.0455***	−0.0259**	−0.0239**
	(−2.76)	(−2.34)	(−2.41)
Observations	3265	3265	3265
Adjusted R-squared	0.860	0.859	0.859
Control	YES	YES	YES
City FE	YES	YES	YES
Year FE	YES	YES	YES

Robust t-statistics in parentheses***p < 0.01, **p < 0.05, *p < 0.1.

China’s vast territory and differences in economic development, institutional environment and resource endowment among regions may lead to the effects of CRexpress on the urban-rural income gap. Table 6 presents the impact of CRexpress on the urban-rural income gap based on three common methods of regional division. Columns (1) to (3) show grouping regressions for the eastern, central, and western regions based on economic development levels. The regression coefficients show that, compared to cities without CRexpress, the urban-rural income gap in cities with CRexpress in the eastern and central regions has narrowed by 8.91% and 3.58%, respectively. However, the urban-rural income gap in cities with CRexpress in the western regions has widened compared to cities without CRexpress by 5.27%. Columns (4) to (5) present grouping regressions for coastal and inland areas. The results suggest that CRexpress has significantly narrowed the urban-rural income gap in coastal areas, but has no significant impact on the urban-rural income gap in inland cities. Columns (6) to (7) show grouping regressions based on China’s traditional population geography dividing line (Hu Huanyong Line). The regression results also manifest that CRexpress has significantly narrowed the urban-rural income gap in the east, but has no significant impact on the urban-rural income gap in the central and western regions. Overall, the effects of CRexpress in narrowing the urban-rural income gap, as shown by the three main methods of dividing China’s regional economy in current academic circles, are larger in the east compared to the central and western regions.

**Table 6:** Regional division of China.

VARIABLES	(1)	(2)	(3)	(4)	(5)	(6)	(7)
	Eastern	Central	Western	Coastal	Inland	Eastern Front	Western Front
	gap	gap	gap	gap	gap	gap	gap
CRexpress	−0.0891***	−0.0358**	0.0527**	−0.0835***	−0.0078	−0.0587***	0.0082
	(−4.97)	(−2.37)	(2.63)	(−4.85)	(−0.63)	(−5.14)	(0.29)
Observations	1257	1292	669	1360	1858	2569	649
Adjusted R-squared	0.803	0.848	0.900	0.844	0.856	0.867	0.865
Control	YES	YES	YES	YES	YES	YES	YES
City FE	YES	YES	YES	YES	YES	YES	YES
Year FE	YES	YES	YES	YES	YES	YES	YES

Robust t-statistics in parentheses ***p < 0.01, **p < 0.05, *p < 0.1.

The above results imply that the industrial development in the central and western regions lags behind the improvement of international trade freight channels. In 2020, the GDP of the secondary industry in eastern, central and western China accounted for 54.07%, 25.5% and 20.4% of the country respectively, that is, China’s secondary industry is still mainly concentrated in the eastern regions, and the ability to undertake industrial transfer in the central and western regions needs to be further strengthened. The above has verified that the secondary industry is the channel through which the CRexpress affects the urban-rural income gap, so it is logical that the CRexpress has a greater effect on the eastern regions.

But this raises the question, in the face of the opening dividends brought by the CRexpress, why did the western and central regions, which are geographically closer to the European market, fail to replicate the “myth” of industrial development in the eastern regions when China joined the WTO in the past? In other words, at the current stage of development, what factors have weakened the ability of the central and western regions to undertake industrial transfer? The possible reason is that the flow of factors closely related to industrial transfer is not only subject to transportation conditions, but also to regional institutional environment [37]. During the sample period, the mean values of the institutional environment in the eastern, central and western regions were 8.02, 6.17 and 5.51, respectively, showing an obvious trend of high in the east and low in the west. The disadvantage of the institutional environment in the central and western regions restricts the free flow of factors such as labor and capital, which is neither conducive to the development of local industries nor to absorbing local rural labor for employment nearby. The previous article also verified the positive moderating effect of the institutional environment, that is, the institutional environment strengthened the effect of narrowing the urban-rural income gap, which can be mutually confirmed with the regression results of regional heterogeneity. Namely, the transportation cost advantages of the central and western regions are offset by the disadvantages of the institutional environment, so the effect of the CRexpress on the eastern regions is even greater.

In order to make the above explanation more robust, a more direct way is to examine the labor mobility represented by migrant workers, because industrial development and labor mobility are closely related. According to the monitoring reports of migrant workers issued by the National Bureau of Statistics over the years, on the whole, the flow trend of migrant workers is from west to east. Taking 2020 as an example, the numbers of migrant workers absorbed in the eastern, central and western regions are 151.32 million, 62.27 million and 62.79 million respectively. From a local point of view, the flow of migrant workers shows obvious heterogeneity. Fig. 5 shows the flow of migrant workers divided by the place of export. The eastern regions have the advantages of a thick labor market due to the developed industry. Migrant workers mainly flow within the province, while migrant workers in the central and western regions tend to move across provinces. The eastern regions have the industrial first-mover advantage, and the institutional environment is better than that of the central and western regions, so it is very attractive to migrant workers who flow across provinces in the central and western regions.

**Fig. 4:**
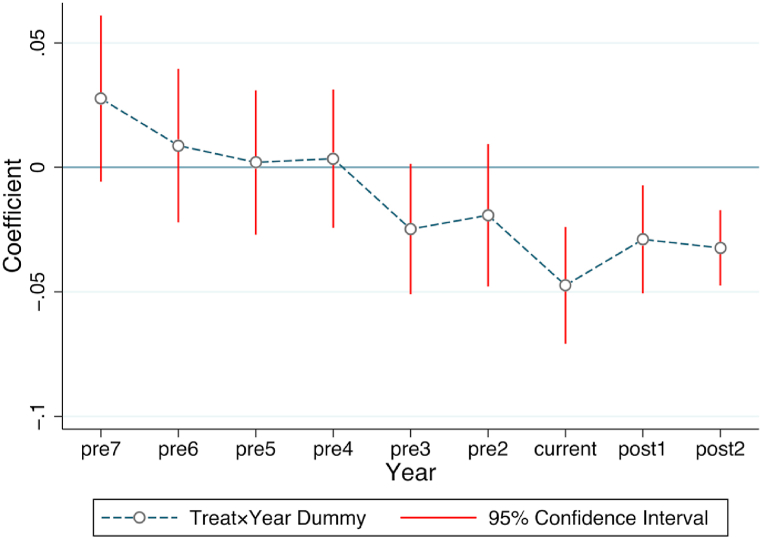
Parallel trend test.

**Fig. 5:**
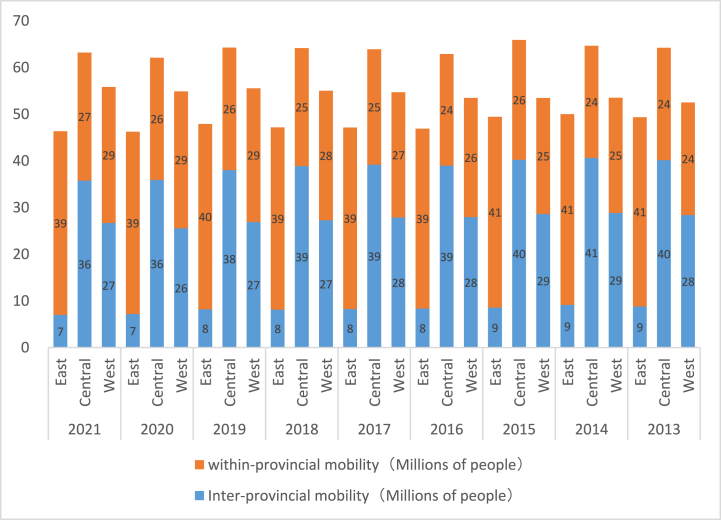
Flow of rural migrant workers (divided by place of export of migrant workers).

It is worth noting that the flow of rural labor will lead to the distribution effect of migrant workers' remittances [46]. Moreover, the distribution effect of migrant workers' remittances on urban and rural income is greater for local mobility than cross-regional mobility,^1^ while the flow of migrant workers in the eastern regions is dominated by local mobility. This will lead to the effect of CRexpress narrowing the urban-rural income gap being greater in the eastern region,^2^ that is, migrant workers' remittances are a potential channel that affects the urban-rural income gap. If the channel of migrant workers' remittance is established, it will be a good evidence. In order to verify this conjecture, in the sixth part, this paper will further analyze the income structure of urban and rural residents in combination with labor mobility, so as to verify the potential channel of migrant workers' remittances.

## 6 Further analysis: decomposition of urban and rural residents' income

This paper will examine the income structure of urban and rural residents in this part. If the potential channel of migrant workers' remittances described above does exist, then the CRexpress will narrow the urban-rural income gap mainly through the transfer of net income. The rationale for this is that transfer income includes various government subsidies and remittances from migrant workers, and government subsidies have uniform standards, so variations in transfer income can be equivalent to variations in remittances from migrant workers.

### 6.1 The flow characteristics of Chinese migrant workers and the adjustment of statistical caliber

**Fig. 6:**
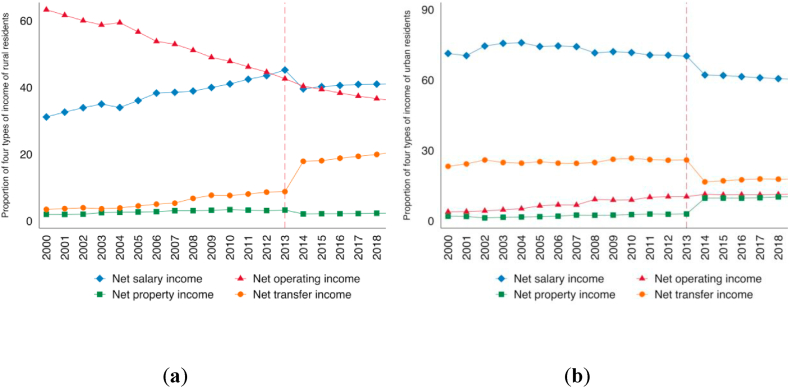
Resident income structure: (a) Trends in four types of income of rural residents; (b) Trends in four types of income of urban residents.

Chinese migrant workers have two key characteristics: mobility and the remittances they send back to their rural families. Mobility is reflected in two ways: in terms of employment, it is from agriculture to non-agricultural industries; in terms of geographical location, it is not only from rural to urban areas, but also from local mobility to cross-regional mobility (from the central and western inland areas to the eastern seaboard). Corresponding to the flow of migrant workers is the remittance of migrant workers. Although migrant workers work in other places, they still maintain close contact with their rural families. It is common for migrant workers to send back a portion of their income to their rural families, and this is often the main purpose of their migrant work [47]. Migrant workers' remittances have become a major source of income for rural families in China. According to a survey by the People’s Bank of China on the employment distribution and income of migrant workers in 2005, the income of migrant workers accounted for about 65% of the total income of migrant households. And more than half of the income of migrant workers was sent back to their hometowns. Migrant workers' remittances represent a transfer payment from migrant workers to rural households for a portion of their urban migrant income. The income of urban and rural residents in China is divided into net operating in-come, net wage income, net property income, and net transfer income. The statistical caliber can have an impact on the urban-rural income gap, as shown in Fig. 6a and b.

In 2013, at the point of the conversion from the old to the new statistical caliber, the proportion of rural residents' net property income and the proportion of net operating income showed a stable linear trend. After 2013, the proportion of urban residents' net transfer income dropped sharply, while the proportion of rural residents' net transfer income jumped significantly. This is because, according to the statistical caliber after 2013, migrant workers' remittances are stripped from migrant workers' wages and included in migrant workers' remittances as transfer income/expenditure. Therefore, further analysis of the structural differences in the income of urban and rural residents can help to better understand the impact of the opening of CRexpress on urban and rural income.

### 6.2 Regression analysis of classified income of urban and rural residents

To better understand the structural differences in the income of urban and rural residents and the impact of the opening of CRexpress on these differences, this study uses provincial panel data on the classified income of urban and rural residents from 2003 to 2020. The treatment group in this analysis consists of provinces with cities having opened CRexpress, while the control group consists of provinces without such cities. The model setup is similar to Model 1, with the addition of two-way fixed effects and control variables in columns (1) to (5) in Table 7.

**Table 7:** Regression of total income and classified income of urban and rural residents.

VARIABLES	(1)	(2)	(3)	(4)	(5)
	Total (gap)	Wage (gap)	Operating (gap)	Property (gap)	Transfer (gap)
CRexpress	−0.035*	0.116	0.083	−0.164	−0.469**
	(−1.80)	(1.64)	(0.94)	(−1.14)	(−2.55)
Observations	580	580	580	580	580
Adjusted R-squared	0.934	0.937	0.833	0.817	0.891
Province FE	YES	YES	YES	YES	YES
Year FE	YES	YES	YES	YES	YES
Control	YES	YES	YES	YES	YES

Robust t-statistics in parentheses ***p < 0.01, **p < 0.05, *p < 0.1.

The results of the regression analysis show that the opening of the China-Europe Railway Express (CRexpress) significantly narrows the total income gap between urban and rural residents in the opening provinces by 3.5%, as indicated in column (1) of Table 7. This finding is consistent with the results obtained from the prefecture-level panel data. Columns (2) to (4) demonstrate that the opening of CRexpress has no significant impact on the income gap between urban and rural residents in terms of wages, business operations, and net property income. However, column (5) shows that the opening of CRexpress significantly narrows the gap between the net transfer income of urban and rural residents by 46.9%.

Combined with the previous analysis, from the perspective of the income structure of urban and rural residents, the effect of CRexpress on narrowing the urban-rural income gap is mainly realized through the channel of transfer income (migrant workers' remittances). This actually explains why the effect of narrowing the urban-rural income gap of the CRexpress is greater in the eastern regions from another perspective, thus increasing the robustness of this paper’s conclusions. Migrant workers in the eastern regions tend to move locally, and the income distribution effect of migrant remittances is manifested as a dual effect on urban and rural income in these regions. Therefore, the opening of the CRexpress has a greater impact on narrowing the urban-rural gap in the eastern regions. Moreover, the distribution effect of this transfer income and the mediating effect of the secondary industry also confirm each other, because more rural labor entering the secondary industry will inevitably bring more remittances from migrant workers.

## 7 Conclusions and policy implications

### 7.1 Conclusions

In this study, China’s prefecture-level panel data and provincial-level panel data from 2003 to 2020 were applied to investigate the impact of the CRexpress on the urban-rural income gap in the country. Our main findings are as follows:

First, the opening of CRexpress has significantly narrowed the urban-rural income gap in cities that have opened the train, and this effect has a radiating impact on the cities surrounding those having opened the train. The effect decreases with the increasing distance.

Second, the mechanism analysis shows that CRexpress affects the urban-rural income gap by promoting the development of the secondary industry in open cities.

Third, this effect exhibits significant regional heterogeneity, with the effect in eastern coastal areas being more significant than that in inland areas of the central and western regions. The institutional environment plays a positive moderating role in this process.

Fourth, by decomposing the income of urban and rural residents, it is found that the CRexpress narrowed the urban-rural income gap mainly through its impact on net transfer income.

### 7.2 Policy implications

Based on these conclusions, the following two policy recommendations are proposed:

First, relying on the role of the CRexpress as an international logistics hub, inland areas should give full play to the effect of resource agglomeration and radiation to better integrate into the global industrial chain. The CRexpress is a major logistics and transportation channel for international trade in the regions along the “Belt and Road Initiative”. The distribution of industrial resources in the inland regions, especially those aimed at exports, should align with the spatial arrangement of the CRexpress route and supply nodes. This strategy can enhance the breadth and depth of export-oriented trade and stimulate new economic growth in the inland areas. Additionally, the spatial planning of regional industries should prioritize improving economic and trade relations between the primary supply and transportation nodes along the CRexpress route, with the aim of promoting the coordinated development of industry and trade in the surrounding areas of the cities where CRexpress is available.

Second, local governments should focus on optimizing the institutional environment, improve the level of trade facilitation, and attract high-quality enterprises to invest. The government should focus on guidance, give full play to the leading role of the market, and encourage and support enterprises with outstanding advantages and mature operations to play a greater leading role. In particular, in order to better undertake industrial transfer, governments in inland regions should focus on improving the business environment, attracting investment from high-quality enterprises, and dismantling local protectionism, so that enterprises can survive the fittest under the role of market mechanisms and enhance industrial competitiveness. In addition, the government should enhance service awareness, coordinate relevant functional areas of business to simplify customs procedures, improve customs clearance speed, and provide better institutional guarantees for industrial development.

### 7.3 Limitations of the study and future research

This study focuses on exploring the impact and mechanism of the opening of the CRexpress on the urban-rural income gap, while also delving into its heterogeneity, providing new empirical evidence for evaluating the economic and social impact of the CRexpress. However, it is important to acknowledge that there are some limitations to this study that warrant further exploration in future research.

First, due to the lack of data information, this paper does not consider the impact of the commodity structure of the CRexpress on the urban-rural income gap. A more in-depth analysis can be conducted in subsequent research to address this issue. Secondly, the empirical analysis results demonstrate that the opening of the CRexpress has a more significant effect in narrowing the urban-rural income gap in the eastern region compared to the central and western regions. This finding suggests that the central and western regions' attractiveness to the labor force remains insufficient. Consequently, future research should investigate the impact of the CRexpress on migrant workers' willingness to relocate as an area of further study.

## Funding statement

This work was supported by the National Key R&D Program Project “Demonstration of Characteristic Economic Forest Industry Chain Integration” (Project No.: 2021YFD1000401).

## Author contribution statement

Lei Zhang: Conceived and designed the experiments; Performed the experiments; Analyzed and interpreted the data; Wrote the paper.

Jianghong Wan: Performed the experiments; Wrote the paper.

Amar Razzaq: Contributed reagents, materials, analysis tools or data; Wrote the paper.

Qing Zhang: Analyzed and interpreted the data; Wrote the paper.

Ling Zhou: Sahar Erfanian: Contributed reagents, materials, analysis tools or data.

## Data availability statement

Data will be made available on request.

## Declaration of competing interest

The authors declare that they have no known competing financial interests or personal relationships that could have appeared to influence the work reported in this paper

## Footnotes

1 If migrant workers flow locally, the distribution effect of migrant workers' remittances is two-way. Urban residents increase transfer expenditures, and rural residents increase transfer income. If migrant workers move across regions, the impact of transfer income/expenditure will be one-way, which will increase the transfer expenditure of urban residents in the input area and increase the transfer income of rural residents in the export area. Obviously, the flow of migrant workers in the region is more conducive to narrowing the urban-rural income gap.

2 The stacking chart of the flow of migrant workers by export location (Fig. 4) shows that the total number of migrant workers in the eastern region decreased slightly, and the proportion of internal flow was higher, and migrant workers were more inclined to seek jobs in the province; The total number of migrant workers in the central region is the highest and relatively stable, and the proportion of inter-provincial mobility is slightly higher. Migrant workers are more inclined to seek higher wages in the eastern developed regions; The total number of migrant workers in the western region is between the eastern and central region, showing a slow growth trend. This reflects the general flow of migrant workers from west to east, and is also consistent with the level of economic development in the east, middle and west of China.
